# Regulatory Dendritic Cells Induced by Bendamustine Are Associated With Enhanced Flt3 Expression and Alloreactive T-Cell Death

**DOI:** 10.3389/fimmu.2021.699128

**Published:** 2021-06-24

**Authors:** Megan S. Molina, Emely A. Hoffman, Jessica Stokes, Nicole Kummet, Kyle A. Smith, Forrest Baker, Tiffany M. Zúñiga, Richard J. Simpson, Emmanuel Katsanis

**Affiliations:** ^1^ Department of Pediatrics, University of Arizona, Tucson, AZ, United States; ^2^ Department of Immunobiology, University of Arizona, Tucson, AZ, United States; ^3^ Department of Molecular & Cellular Biology, University of Arizona, Tucson, AZ, United States; ^4^ Department of Physiology, University of Arizona, Tucson, AZ, United States; ^5^ Department of Nutritional Sciences, University of Arizona, Tucson, AZ, United States; ^6^ The University of Arizona Cancer Center, Tucson, AZ, United States; ^7^ Department of Medicine, University of Arizona, Tucson, AZ, United States; ^8^ Department of Pathology, University of Arizona, Tucson, AZ, United States

**Keywords:** dendritic cell, Flt3, bendamustine, Alloreactivity, Regulatory DC

## Abstract

The growth factor Flt3 ligand (Flt3L) is central to dendritic cell (DC) homeostasis and development, controlling survival and expansion by binding to Flt3 receptor tyrosine kinase on the surface of DCs. In the context of hematopoietic cell transplantation, Flt3L has been found to suppress graft-versus-host disease (GvHD), specifically *via* host DCs. We previously reported that the pre-transplant conditioning regimen consisting of bendamustine (BEN) and total body irradiation (TBI) results in significantly reduced GvHD compared to cyclophosphamide (CY)+TBI. Pre-transplant BEN+TBI conditioning was also associated with greater Flt3 expression among host DCs and an accumulation of pre-cDC1s. Here, we demonstrate that exposure to BEN increases Flt3 expression on both murine bone marrow-derived DCs (BMDCs) and human monocyte-derived DCs (moDCs). BEN favors development of murine plasmacytoid DCs, pre-cDC1s, and cDC2s. While humans do not have an identifiable equivalent to murine pre-cDC1s, exposure to BEN resulted in decreased plasmacytoid DCs and increased cDC2s. BEN exposure and heightened Flt3 signaling are associated with a distinct regulatory phenotype, with increased PD-L1 expression and decreased ICOS-L expression. BMDCs exposed to BEN exhibit diminished pro-inflammatory cytokine response to LPS and induce robust proliferation of alloreactive T-cells. These proliferative alloreactive T-cells expressed greater levels of PD-1 and underwent increased programmed cell death as the concentration of BEN exposure increased. Alloreactive CD4^+^ T-cell death may be attributable to pre-cDC1s and provides a potential mechanism by which BEN+TBI conditioning limits GvHD and yields T-cells tolerant to host antigen.

## Introduction

Fms-like tyrosine kinase 3 (Flt3) (aka CD135, Flk2, STK1) is a receptor tyrosine kinase that binds the growth factor Flt3 Ligand (Flt3L) (1–5). Flt3 is expressed by early hematopoietic cells and controls their survival and expansion (3, 4, 6, 7). Flt3 expression is lost as hematopoietic precursors differentiate, but expression is maintained on dendritic cells (DCs) through their terminal differentiation (8, 9). Flt3 signaling is crucial to the homeostasis and development of steady state DCs (3, 10–17). Given the critical role of DCs in graft-versus-host disease (GvHD) (18, 19) and graft-versus-leukemia (GvL) (20), Flt3L has been investigated by numerous groups in the context of hematopoietic stem cell transplantation (HSCT). Administration of Flt3L to recipients *prior* to transplant significantly reduces GvHD, an effect largely attributed to increased numbers of host CD8α^+^ type 1 conventional DCs (cDC1s) capable of inducing clonal deletion of alloantigen-specific T-cells (21–23).

Previous work from our laboratory using murine bone marrow transplantation (BMT) models found that bendamustine (BEN) supplemented with total body irradiation (TBI) conditioning results in significantly reduced GvHD and improved survival compared to cyclophosphamide (CY)+TBI, the standard regimen used in cases of acute lymphoblastic leukemia (ALL) (24–27). BEN is a cytotoxic alkylating agent with diverse immunomodulatory properties (24–26, 28–33). Importantly, BEN+TBI conditioning yields donor T-cells that are tolerant to host, while preserving T-cell-dependent GvL (26). BEN+TBI also results in a more favorable host DC composition at the time of transplant, with increased frequencies of cDC1s, most substantially pre-cDC1s (27). Host DCs from BEN-treated mice also display greater Flt3 expression compared to CY-treated DCs (27). It remains unclear if increased Flt3 expression is a direct effect of BEN. Given the clear advantage of enhanced Flt3 signaling in host DCs in the context of transplantation, this warranted further investigation. Moreover, it is not understood whether enhanced Flt3 expression alters DC development or function in the same manner as administration of exogenous Flt3L.

Here we investigate the ability of BEN to directly induce increased Flt3 expression in murine bone marrow (BM) progenitors and DCs, and examine the effect of BEN exposure on dendropoiesis in murine and human DCs *in vitro*. We further investigate how murine DCs exposed to BEN mature in response to TLR activation and stimulate alloreactive T-cell responses. Overall, our results demonstrate that BEN elicits a regulatory program in DCs, associated with increased Flt3 signaling. This “regulatory” program is exemplified by increased expression of inhibitory co-stimulatory molecules (PD-L1), a minimal pro-inflammatory response to lipopolysaccharide (LPS) stimulation, and robust activation-induced death of alloreactive CD4^+^ T-cells. This work highlights the capacity of Flt3L-driven DCs to regulate alloreactive CD4^+^ T-cell responses in a way that is highly advantageous for GvHD and may preserve GvL by sparing alloreactive CD8^+^ T-cells.

## Methods

### Mice

All strains of mice used (BALB/c and C57BL/6) were age-matched 6-10-week-old females purchased from The Jackson Laboratory. Mice were housed in specific pathogen-free conditions and cared for according to the guidelines of the University of Arizona’s Institutional Animal Care and Use Committee.

### Drug Preparation and Administration

BEN (SelleckChem) was reconstituted and diluted for *in vivo* administration as previously described (24–27). AC220 (SelleckChem) and JSI-124 (Santa Cruz Biotechnology) were reconstituted in DMSO (Sigma-Aldrich). For *in vitro* studies, stock solutions of drugs were diluted in complete media (CM) consisting of RPMI-1640 with 10% FBS, 1% Sodium Pyruvate, 1% MEM NEAA, and 100 U/mL penicillin-streptomycin to their final concentrations.

### Murine Bone Marrow-Derived DCs (BMDCs)

Murine bone marrow (**BM**) cells were collected, red blood cells were lysed with Pharm Lyse (BD Biosciences), and 3x10^6^ BM cells were plated per well in 6-well plates at a concentration of 10^6^/mL. BM was cultured at 37°C and 5% CO_2_ in CM containing 200 ng/mL of rhFlt3L (Miltenyi Biotec) with or without drugs (bendamustine, AC220, or JSI-124). After 4 hours of culture, all media were washed out, BM cells were washed with PBS and again cultured in CM containing 200 ng/mL of rhFlt3L. Culture media was replenished on day 3 and 5. LPS (Sigma-Aldrich) was added on day 5 of culture for 18 hours at a final concentration of 1 μg/mL. Individual wells of BMDCs were collected on day 6.

### Absolute Counts and Viability

BMDCs were resuspended in PBS and analyzed by MACSQuant Analyzer 10 (Miltenyi Biotec) to determine absolute counts and viability by Propidium Iodide staining.

### Flow Cytometry

Cells were washed in flow buffer (PBS with 0.5% FBS), incubated with anti-mouse or anti-human Fc Block (Thermo Fisher Scientific), and flow cytometry was performed as previously reported (24–27, 34). Intracellular staining of human moDCs was performed using TruePhos Perm Buffer (Biolegend). All antibodies used for flow cytometry are listed in **Table 1**. Fluorescence data were collected using an LSRFortessa cell analyzer (BD Biosciences) and analyzed using FlowJo 2 (Tree Star). Total DCs were defined as CD11c^+^. Plasmacytoid DCs (pDCs) were defined as CD11c^+^B220^+^. Conventional DCs (cDCs) were defined as CD11c^+^ B220^-^. Type 1 conventional DCs (cDC1s) were defined as CD11c^+^B220^-^CD8α^+^ and CD11c^+^B220^-^CD103^+^. Type 2 conventional DCs (cDC2s) were defined as CD11c^+^B220^-^SIRPα^+^. Pre-cDC1s were defined as CD11c^+^B220^-^CD24^high^CD8α^-^. Pre-cDC2s were defined as CD11c^+^B220^-^SIRPα^+^CD24^mid^.

**Table 1:** Antibodies used for flow cytometry.

Antibody	Clone(s)	Vendor
Anti-mouse B220 Brilliant Violet 510	RA3-6B2	Biolegend
Anti-mouse CCL2 PE	2H5	Biolegend
Anti-mouse CCL5 PE-Cyanine7	2E9	Biolegend
Anti-mouse CD4 APC/Cy7	GK1.5	Biolegend
Anti-mouse CD8α PE-Cyanine7	53-6.7	Thermo Fisher
Anti-mouse CD11c FITC	N418	Miltenyi Biotec
Anti-mouse CD11c VioBlue	REA754	Miltenyi Biotec
Anti-mouse CD24 Pacific Blue	M1/69	Biolegend
Anti-mouse CD24 PE-Dazzle 594	M1/69	Biolegend
Anti-mouse CD69 PE/Cyanine5	H1.2F3	Thermo Fisher
Anti-mouse CD70 PerCP-eFluor710	FR70	Thermo Fisher
Anti-mouse CD80 APC	16-10A1	Biolegend
Anti-mouse CD86 AlexaFluor700	GL-1	Biolegend
Anti-mouse CD103 PE	2E7	Thermo Fisher
Anti-mouse CD135 PE-CF594	A2F10.1	BD Biosciences
Anti-mouse H2Kb PerCP-eFluor710	AF6-88.5.5.3	Thermo Fisher
Anti-mouse ICOS VioGreen	REA192	Miltenyi Biotec
Anti-mouse ICOSL PE	HK5.3	Biolegend
Anti-mouse IL-6 APC	MP5-20F3	Biolegend
Anti-mouse IL-10 APC-Cyanine7	JES5-16E3)	Biolegend
Anti-mouse IDO-1 AlexaFluor 647	2E2/IDO1	Biolegend
Anti-mouse PD-1 APC	29F.1A12	Biolegend
Anti-mouse PD-L1 PE/Dazzle594	10F.9G2	Biolegend
Anti-mouse PIR-B APC	10-1-PIR	Thermo Fisher
Anti-mouse SIRPα APC-Cyanine7	P84	Biolegend
Anti-mouse TIM-3 PE	REA602	Miltenyi Biotec
Anti-mouse TNFα Brilliant Violet 510	MP6-XT22	Biolegend
Anti-human AXL PE-Cyanine7	DS7HAXL	Thermo Fisher
Anti-human BDCA1 PE-Vio615	REA694	Miltenyi Biotec
Anti-human BDCA3 APC-Vio770	REA774	Miltenyi Biotec
Anti-human BDCA3 Brilliant Violet 421	M80	Biolegend
Anti-human CD11c AlexaFluor 488	3.9	Biolegend
Anti-human CD14 Brilliant Violet 421	MPHIP9	BD Biosciences
Anti-human Clec9a PE	8F9	Biolegend
Anti-human Lineage (CD3/14/19/20/56) Cocktail APC	UCHT1; HCD14; HIB19; 2H7; HCD56	Biolegend
Anti-human STAT3 Phospho(Tyr705) PerCP/Cyanine5.5	13A3-1	Biolegend
Isotype BV510 Rat IgG1,k	RTK2071	Biolegend
Isotype APC Rat IgG1	RTK2071	Biolegend
Isotype PE Armenian Hamster IgG	HTK888	Biolegend
Isotype PE-Cyanine7 Mouse IgG2b,k	MPC-11	Biolegend
Isotype APC-Cyanine7 Rat IgG2b	RTK4530	Biolegend

### ELISAs

Cytokines in culture supernatants were measured with ELISA kits (R&D Systems).

### Intracellular Cytokine Staining

FL-BMDCs were LPS-activated on day 6 for 3-4 hours. Protein transport inhibitors GolgiStop (Thermo Fisher) and GolgiPlug (Thermo Fisher) were incubated with FL-BMDCs for 4-6 hours. After Fc block, FL-BMDCs were fixed and stained using Fixation Buffer (Biolegend) and Intracellular Staining Perm Wash Buffer (Biolegend). Antibodies used for flow cytometry are listed in **Table 1**.

### Mixed Leukocyte Reaction (MLR)

Unstimulated FL-BMDCs were counted and enriched for live cells using EasySep Dead Cell Removal (Annexin V) kit (STEMCELL Technologies). Allogeneic T-cells were isolated from the spleens of naïve C57BL/6 mice using the Pan T-cell isolation kit II (Miltenyi Biotec). Purified T-cells were stained with CellTrace Violet (Invitrogen). Live FL-BMDCs were co-cultured with allogeneic T-cells at a ratio of 1:10 and incubated at 37°C with 7.5% CO_2_. T-cells were stimulated with CD3/CD28 DynaBeads (Thermo Fisher Scientific) as a positive control. After 16-24 hours, rIL-2 (PeproTech) was added to each well at a final concentration of 50 IU/mL. After 3-4 days of co-incubation flow cytometry was performed, and data were analyzed using Modfit software (Verity Software House) to determine the proliferation index (PI) of H2K^b+^ T-cells. T-cell death was determined using Propidium Iodide Ready Flow Reagent (Invitrogen).

### Human Monocytic-DCs

Healthy human volunteers were recruited as part of an institutional review board (IRB)-approved research protocol. Our protocol for generating human monocytic-DCs (moDC) was adapted from previously reported protocols (35–37). Peripheral blood was collected and whole blood was diluted 1:1 with PBS, layered on top of Ficoll (GE Healthcare Life Sciences), and then centrifuged per the manufacturer’s recommendation. CD14^+^ monocytes were isolated using CD14^+^ MicroBeads (Miltenyi Biotec) with >97% purity (data not shown), counted, and then re-suspended in RPMI-14 containing 10% FBS, 10% antibiotic-antimycotic solution (ThermoFisher), 500 U/mL rhIL-4 (PeproTech), 800 U/mL rhGM-CSF (PeproTech), and 100 ng/mL rhFlt3L (Miltenyi Biotec). Monocytes were plated into a 6-well plate with 1-1.5x10^6^ monocytes per well, the indicated concentration of BEN, and then cultured at 37°C and 5% CO_2_. After 4 hours, all BEN-containing media was washed out, cells were washed with PBS and cultured again in the same media at 37°C and 5% CO_2_. Media was replenished on day 3 of culture, and moDCs were collected on day 5 of culture for flow cytometry.

### qRT-PCR

Samples were saved in PBS and RNAlater (Invitrogen), mRNA was isolated using RNeasy Kit (Qiagen) and then reverse transcribed into cDNA using iScripts reverse transcription supermix kit (Bio-Rad). Quantitative rtPCR was performed using Sso Advanced universal probes supermix (Bio-Rad) on a LightCycler 96 thermocycler (Roche) named Laurel. The appropriate concentration of cDNA was titrated for each TaqMan probe (Applied Biosystems), listed in **Table 2**. The 2^-ΔΔCT^ method was used to analyze gene expression levels, normalized for GAPDH expression, as previously described (38, 39).

**Table 2:** Primers used for qRT-PCR.

Target Gene	Taqman Assay ID	Concentration of cDNA used
Mouse Akt1	Mm01331626_m1	5 ng
Mouse Csf2ra	Mm00438331_g1	5 ng
Mouse Csf2rb	Mm00655745_m1	5 ng
Mouse Csf3r	Mm00438334_m1	10 ng
Mouse Flt3	Mm00439016_m1	20 ng
Mouse GAPDH	Mm99999915_g1	2 ng
Mouse Spi1 (PU.1)	Mm00488140_m1	5 ng
Mouse STAT3	Mm01219775_m1	10 ng
Human Akt1	Hs00178289_m1	2 ng
Human GAPDH	Hs02786624_g1	2 ng

### Statistical Analysis

One-way ANOVA tests and Dunnett’s multiple comparisons tests were used to determine significance among absolute counts, percent, and MFI expression. Two-way ANOVA tests and Šidák’s multiple comparisons tests were used to determine significance in unstimulated versus LPS-stimulated conditions. P values <0.05 were considered statistically significant.

## Results

### Dose-Dependent Increase in Flt3 Expression on Murine Bone Marrow Cells *In Vivo* Following BEN Administration

**Figure 1:**
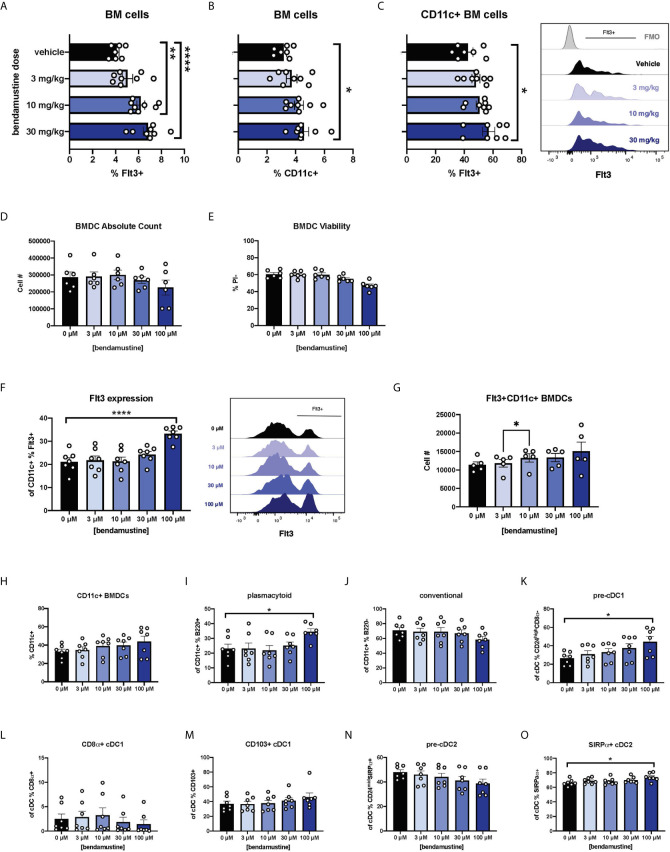
Dose-dependent and concentration-dependent increases in Flt3 expression and skewed DC composition of murine DCs exposed to BEN. **(A–C)** BALB/c mice were i.v. injected with various doses of bendamustine or vehicle and bone marrow (BM) was harvested 48 hours later for analysis by flow cytometry. Data is pooled from 3 independent experiments (n=7-8). **(A)** Mean percent Flt3 expression on total BM cells is shown with SEM. **(B)** The mean percentage of CD11c^+^ DCs within bone marrow is shown with SEM. **(C)** Among CD11c^+^ DCs within bone marrow, mean percent Flt3 expression is shown with SEM. Representative histogram shown (right) with Fluorescence Minus Once (FMO) control. **(D–N**) BALB/c FL-BMDCs were generated following brief exposure to BEN and characterized by flow cytometry. Data is pooled from 3 independent experiments (n=6-7). **(D)** Mean absolute cell number and **(E)** percent viable (Propidium Iodide-) cells are shown with SEM. **(F)** Mean percent Flt3 expression among CD11c^+^ FL-BMDCs is shown with SEM (left) and representative histograms (right). **(G)** Mean absolute cell number of Flt3^+^ CD11c^+^ FL-BMDCs is shown with SEM. **(H–N)** Mean percent with SEM of murine DC lineages including **(H)** total CD11c^+^, **(I)** plasmacytoid DCs (CD11c^+^B220^+^), **(J)** conventional DCs (CD11c^+^B220^-^), **(K)** pre-cDC1s (CD11c^+^B220^-^CD24^high^CD8α^-^), **(L)** CD8α^+^ cDC1s (CD11c^+^B220^-^CD8α^+^), **(M)** CD103^+^ cDC1s (CD11c^+^B220^-^CD103^+^), **(N)** pre-cDC2s (CD11c^+^B220^-^ CD24^mid^SIRPα^+^), and **(O)** SIRPα^+^ cDC2s (CD11c^+^B220^-^SIRPα^+^). One-way ANOVA and Dunnett’s multiple comparisons test were used to determine significance among groups. *P < 0.05, **P < 0.01, ****P < 0.0001.

We first sought to determine whether our previous report of increased Flt3 expression *in vivo* was a direct effect of BEN. Mice were given various doses of BEN or vehicle and bone marrow (BM) was collected 48 hours later, reproducing the timing used in our previously published dosing regimens (25–27). There was an anticipated decrease in absolute counts as the dose of BEN increased, but we found no loss of viability of BM cells (data not shown). We observed that the percent expression of Flt3 increased in a dose-dependent manner on total BM cells (**Figure 1A**). We also found a dose-dependent increase in the percent of CD11c^+^ DCs within the BM compartment (**Figure 1B**) and Flt3 expression on CD11c^+^ BM cells (**Figure 1C**).

### BEN Exposure Increases Flt3 Expression on Murine DCs *In Vitro*

We next sought to eliminate physiological variables by utilizing *in vitro* bone marrow-derived DC (BMDC) systems (40–45). Murine BM cells were cultured with Flt3L (FL-BMDCs) in the presence of BEN for just 4 hours to more closely mimic clinical exposure to BEN which has a short half-life of ~40 minutes (46). After exposure to various concentrations of BEN (0 μM, 3 μM, 10 μM, 30 μM, or 100 μM) in culture for 4 hours, BM cells were washed in PBS, then cultured again for the remaining 6 days with Flt3L. As expected, we saw a modest decrease in absolute number (**Figure 1D**) and percent viable FL-BMDCs (**Figure 1E**) as the concentration of BEN increased. We also observed a concentration-dependent increase in percent Flt3 expression among total live CD11c^+^ FL-BMDCs (**Figure 1F**). The absolute number of Flt3^+^ CD11c^+^ BMDCs increases as the concentration of BEN increases (**Figure 1G**) suggesting that BEN is not selectively killing Flt3-negative cells.

### BEN Exposure Favors Plasmacytoid, Pre-cDC1, and cDC2 Development

FL-BMDCs generated following 4-hour exposure to BEN were characterized to determine DC composition. As the concentration of BEN increased, the percentage of CD11c^+^ FL-BMDCs trended upward (**Figure 1H**) while the percentage of pDCs significantly increased (**Figure 1I**) and the percentage of cDCs slightly decreased (**Figure 1J**). We observed a concentration-dependent increase in pre-cDC1s (**Figure 1K**), however we do not observe an increase in CD8α^+^ cDC1s (**Figure 1L**) and only a slight trend toward increased CD103^+^ cDC1s (**Figure 1M**). We see a trend toward decreased pre-cDC2s (**Figure 1N**) and an increase in SIRPα^+^ cDC2s (**Figure 1O**). These results largely match our report on BEN’s effect on DC composition *in vivo* (27), indicating that BEN promotes DC development in favor of pDCs, pre-cDC1s, and cDC2s.

### BEN Exposure Alters Co-Stimulatory and Co-Inhibitory Molecule Expression

**Figure 2:**
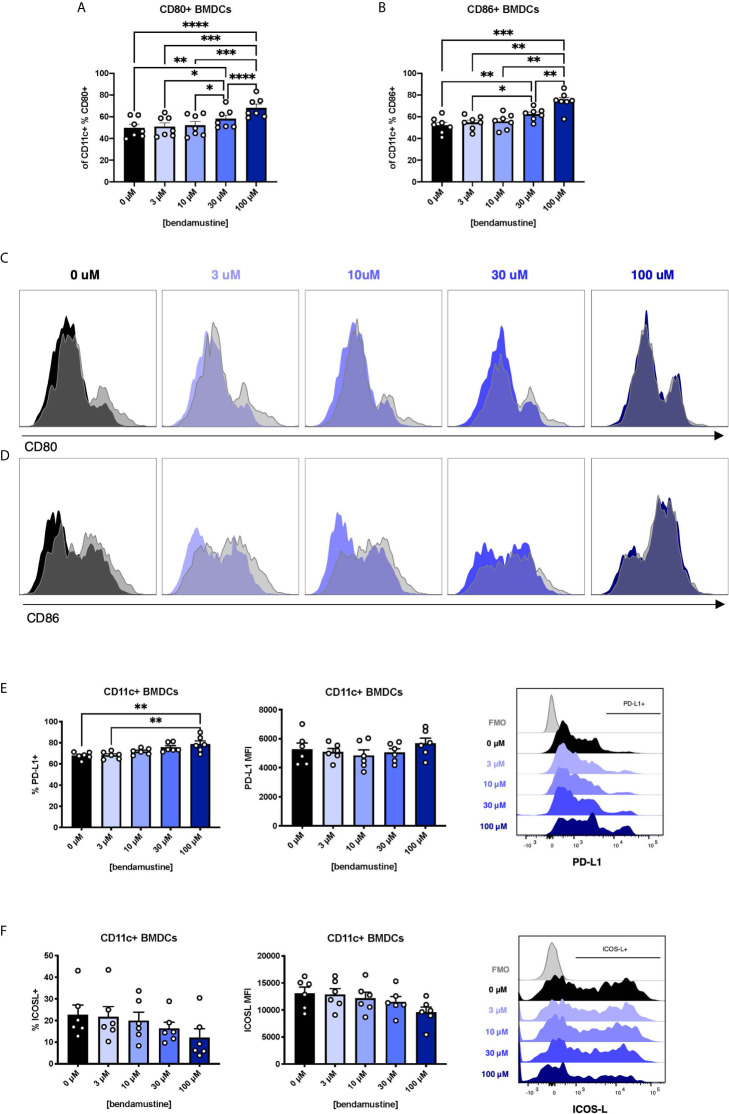
Concentration-dependent increase in CD80, CD86, and PD-L1 expression and dampened response to LPS by murine DCs exposed to BEN. **(A–F)** BALB/c FL-BMDCs were generated following brief exposure to BEN and characterized by flow cytometry. Data is pooled from 3 independent experiments (n=6-7). Mean percent CD80 **(A)** and CD86 **(B)** expression among CD11c^+^ FL-BMDCs is shown with SEM. Representative histograms of CD80 **(C)** and CD86 **(D)** expression on CD11c^+^ FL-BMDCs exposed to the indicated concentration of BEN. Unstimulated condition shown in solid color and corresponding LPS stimulated condition overlaid in gray. **(E)** Mean percent PD-L1 expression (left) and MFI (middle) among unstimulated CD11c^+^ FL-BMDCs shown with SEM, and representative histogram (right). **(F)** Mean percent ICOS-L expression (left) and MFI (middle) among unstimulated CD11c^+^ FL-BMDCs shown with SEM, and representative histogram (right). One-way ANOVA and Dunnett’s multiple comparisons test were used to determine significance among groups. *P < 0.05, **P < 0.01, ***P < 0.001, ****P < 0.0001.

FL-BMDCs are reportedly more steady state-like than GM-BMDCs (42–44). We inquired whether the increased Flt3 expression observed in BEN-exposed FL-BMDCs equated to enhancement of Flt3L-driven steady state features. We assessed B7 molecule expression on FL-BMDCs and found a progressive increase in expression of CD80 (**Figure 2A**) and CD86 (**Figure 2B**) by percent, but not by MFI **(Supplementary Figures 1A, B)**, as the concentration of BEN increases. With 100μM BEN exposure, FL-BMDCs did not exhibit any increase in CD80 or CD86 expression upon LPS stimulation, depicted in histograms with LPS-stimulated DCs overlaid in gray (**Figures 2C, D**) and quantified in **(Supplementary Figures 1C, D)**. Extending our analyses to other co-signaling molecules we demonstrate that the percent expression of PD-L1 significantly increased with higher concentrations of BEN (**Figure 2E**), while the opposite was true with ICOS-L expression (**Figure 2F**). We found no significant changes in expression of CD70, PIR-B, or indoleamine 2,3-dioxygenase **(Supplementary Figures 2A–C)**. All together, we ascertained that BEN-exposed FL-BMDCs are less responsive to LPS stimulation and exhibit greater PD-L1 expression.

### BEN Exposure Inhibits Pro-Inflammatory Cytokine Secretion

**Figure 3:**
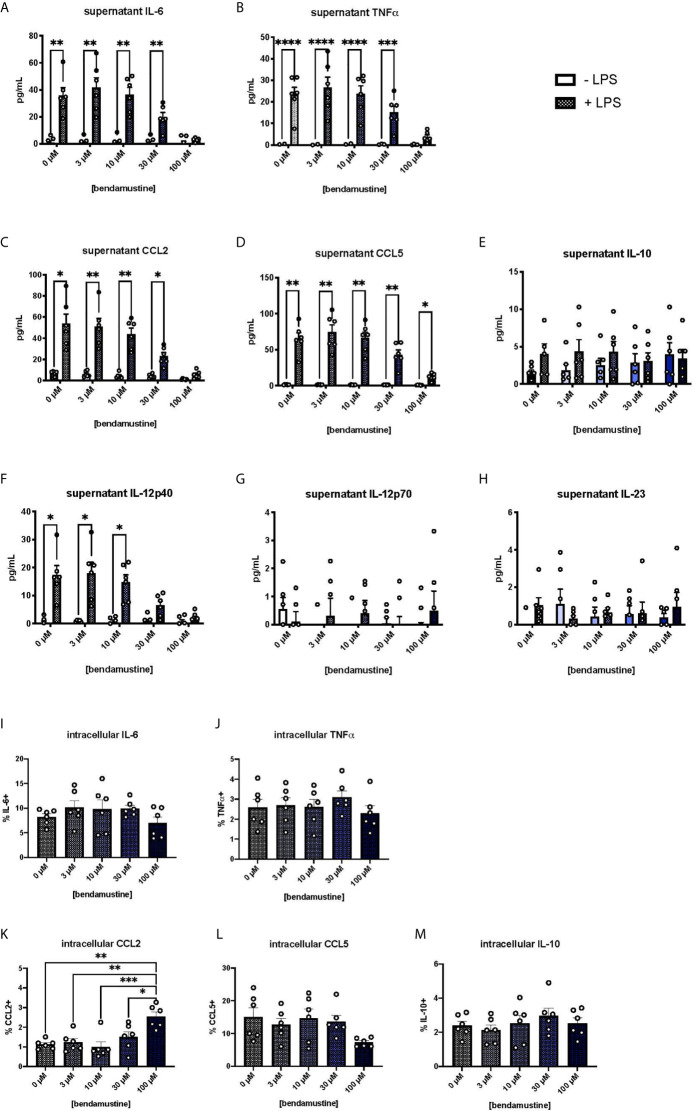
Hindered secretion of pro-inflammatory cytokines in response to LPS by murine DCs exposed to BEN. **(A-H)** BALB/c FL-BMDCs were generated following brief exposure to BEN. With or without 18 hours of LPS stimulation, supernatants were collected for analysis. Data is pooled from 2 independent experiments (n=6). Some values fall below zero, outside of the detectable limits of the assay and outside the axis limits. Mean concentration of **(A)** IL-6, **(B)** TNFα, **(C)** CCL2 (MCP-1), **(D)** CCL5 (RANTES), **(E)** IL-10, **(F)** IL-12p40, **(G)** IL-12p70, and **(H)** IL-23 in supernatants is shown with SEM. Two-way ANOVA and Šidák’s multiple comparisons test were used to determine significance among groups. **(I–M)** Murine BMDCs were generated following brief exposure to BEN. BMDCs were stimulated with LPS for 3-4 hours and treated with protein transport inhibitors prior to intracellular cytokine staining protocol. Gating was set based on FMO and isotype controls. Data is pooled from 2 independent experiments (n=6). Mean percent of **(I)** IL-6^+^, **(J)** TNFα^+^, **(K)** CCL2^+^, **(L)** CCL5^+^, and **(M)** IL-10^+^ FL-BMDCs shown with SEM. One-way ANOVA and Dunnett’s multiple comparisons test were used to determine significance among groups. *P < 0.05, **P < 0.01, ***P < 0.001, ****P < 0.0001.

We next examined pro-inflammatory cytokine secretion by BEN-exposed FL-BMDCs by measuring cytokine concentrations in culture supernatants. Pro-inflammatory cytokines were negligible in unstimulated FL-BMDC cultures. Upon LPS stimulation, control FL-BMDCs (0μM) showed a robust increase in the pro-inflammatory cytokines and chemokines IL-6, TNFα, CCL5, and CCL2 (**Figures 3A–D**). Concentrations of these pro-inflammatory cytokines moderately decreased as the concentration of BEN exposure increased, with a steep drop-off at 100μM (**Figure 3A–D**). However, we did not observe the same effect with the anti-inflammatory cytokine IL-10 (**Figure 3E**). We also found that 100μM BEN exposure significantly hinders secretion of IL-12p40 in response to LPS (**Figure 3F**), though IL-12p70 and IL-23 levels remained very low in all conditions (**Figures 3G, H**). Statistical significance between concentrations of BEN are shown in **(Supplementary Figures 3A–H)**. Intracellular cytokine staining revealed no deficit in intracellular levels of IL-6, TNFα, CCL5, or IL-10, and significantly increased CCL2 (**Figures 3I–M**) in 100μM BEN-exposed FL-BMDCs. These results indicate that BEN exposure diminished secretion of pro-inflammatory cytokines by FL-BMDCs in response to LPS.

### BEN-Exposed FL-BMDCs Induce Allogeneic CD4+ T-Cell Proliferation Followed by Cell Death

**Figure 4:**
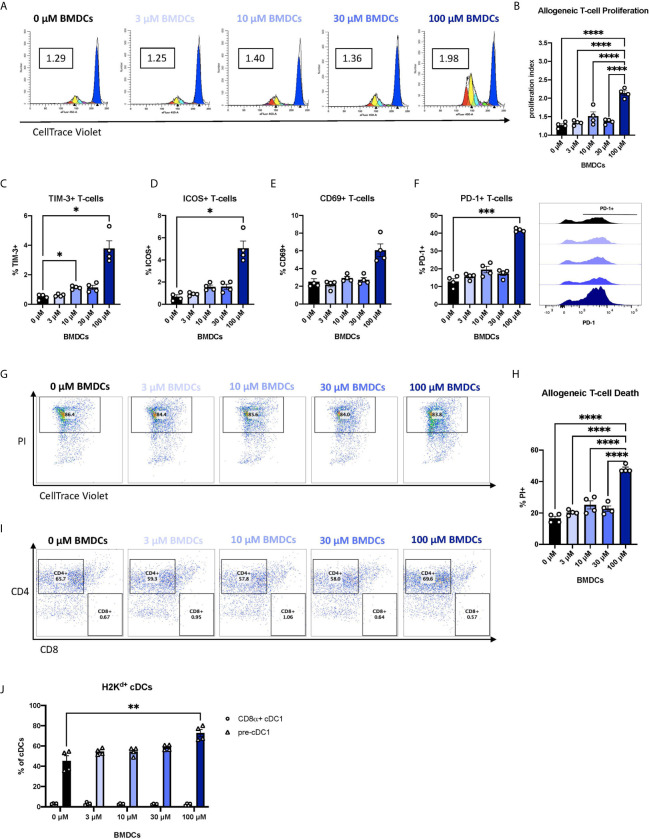
Murine DCs exposed to BEN induce robust proliferation of alloreactive T-cells and expression of PD-1, followed by T-cell death. BALB/c FL-BMDCs were generated following brief exposure to BEN and co-cultured with C57BL/6 CellTrace-stained allogeneic T-cells at a ratio of 1:10. Data shown is representative of 2 independent experiments (n=4). **(A)** Representative histograms generated by ModFit software to quantify T-cell proliferation on day 3 as a proliferation index (PI) (boxed value) in response to stimulation with FL-BMDCs exposed to the indicated concentration of BEN (Gated on H2K^b+^ to exclude DCs from analysis). **(B)** Mean proliferation index on day 3 of co-culture shown with SEM. **(C–E)** Mean percent expression of **(C)** TIM-3, **(D)** ICOS (CD278), and **(E)** CD69 on H2K^b+^ allogeneic T-cells on day 3 shown with SEM. **(F)** Mean percent expression of PD-1 on H2K^b+^ allogeneic T-cells on day 3 shown with SEM, and representative histograms (right). **(G)** Representative flow cytometry plots indicating the percent of dead (PI^+^) T-cells within the proliferative fraction (Gated on H2K^b+^CellTrace^low^). **(H)** Allogeneic T-cell death on day 4 of the assay shown as mean percent of all allogeneic T-cells in culture with SEM. One-way ANOVA and Dunnett’s multiple comparisons test were used to determine significance among groups. **(I)** Representative flow cytometry plots indicating the percentages of CD4^+^ and CD8^+^ T-cells among dead allogeneic T-cells on day 4 (Gated on H2K^b+^CellTrace^low^PI^+^). **(J)** Mean percent of CD8α^+^ cDC1s (circles) and pre-cDC1s (triangles) among H2K^d+^CD11c^+^B220^-^ FL-BMDCs in co-culture with allogeneic T-cells on day 3 shown with SEM. Two-way ANOVA and Šidák’s multiple comparisons test were used to determine significance among groups. *P < 0.05, **P < 0.01, ***P < 0.001, ****P < 0.0001.

We next asked whether the changes in co-signaling molecule expression and pro-inflammatory response of BEN-exposed FL-BMDCs affects alloreactive T-cell responses. Enriched live FL-BMDCs were co-cultured with CellTrace-stained allogeneic T-cells in a mixed leukocyte reaction (MLR). Allogeneic T-cells stimulated with BEN-exposed FL-BMDCs exhibited significantly greater allogeneic T-cell proliferation (**Figure 4A**), quantified by proliferation index (**Figure 4B**), on day 3. Most proliferation was among CD4^+^ T-cells (60-70%), with CD8^+^ T-cells comprising <5% of proliferated T-cells and the remainder being double negative for CD4 and CD8 **(Supplementary Figures 4A–C)**. We further interrogated the phenotype of the alloreactive T-cells by measuring expression of various markers of T-cell activation, anergy, or exhaustion. 100μM BEN-exposed FL-BMDCs induced greater expression of TIM-3, a marker of T-cell exhaustion, as well as ICOS and CD69, markers of T-cell activation (**Figures 4C–E**). FL-BMDCs previously exposed to 100μM of BEN also induced significantly greater expression of PD-1 (**Figure 4F**). PD-1 is a negative regulator of immune responses and plays a central role in generating peripheral tolerance by promoting programmed cell death of antigen-specific T-cells. We next measured alloreactive T-cell death, which is reportedly induced by Flt3L-expanded DCs (23). As activated T-cells are known to upregulate phosphatidylserine, Annexin V was not used to quantify alloreactive T-cell death. On day 4 of co-culture, we first gated on proliferated, allogeneic (CellTrace^low^H2K^b+^) T-cells and then quantified T-cell death by PI-positive staining (**Figure 4G**). When we calculate T-cell death as a percentage of all allogeneic T-cells in culture we find that those stimulated with 100μM BEN-exposed FL-BMDCs exhibited significantly greater T-cell death, with 50% of all T-cells dead on day 4 (**Figure 4H**), most of which were CD4^+^ T-cells (**Figure 4I**). T-cell death induced by 100μM BEN-exposed FL-BMDCs was significantly greater than death observed following stimulation with CD3/CD28 beads **(Supplementary Figure 4D)**, which induced greater T-cell proliferation **(Supplementary Figure 4E)**, indicating that cell death was not merely a result of robust T-cell proliferation. In summary, BEN-exposed FL-BMDCs exhibit an enhanced ability to induce alloreactive T-cell proliferation and cell death.

Previous reports of programmed cell death of alloreactive T-cells have attributed the effect to CD8α^+^ DCs (23), yet our FL-BMDC system yields fewer than 5% CD8α^+^ cDC1s (**Figure 1L**). We observe robust frequencies of their immediate precursor, pre-cDC1s (**Figure 1K**), and asked whether pre-cDC1s were maturing into CD8α^+^ cDC1s in co-culture to induce T-cell death. We demonstrate that by day 3 of co-culture, pre-cDC1s do not mature into CD8α^+^ cDC1s and remain the predominant population of cDCs (**Figure 4J**). Compared to the FL-BMDC proportions plated on day 0, quantified in **Figures 1H–N**, pre-cDC1s effectively double in percentage, perhaps due to their enhanced life-span compared to CD8α^+^ cDC1 (47). This indicates that CD8α^+^ cDC1 may not be the only DC subset capable of inducing deletion of alloreactive T-cells and may signify a previously unknown capability of pre-cDC1s to mitigate alloreactive T-cell responses.

### Inhibitor of Flt3 Elicits Similar DC Phenotype

**Figure 5:**
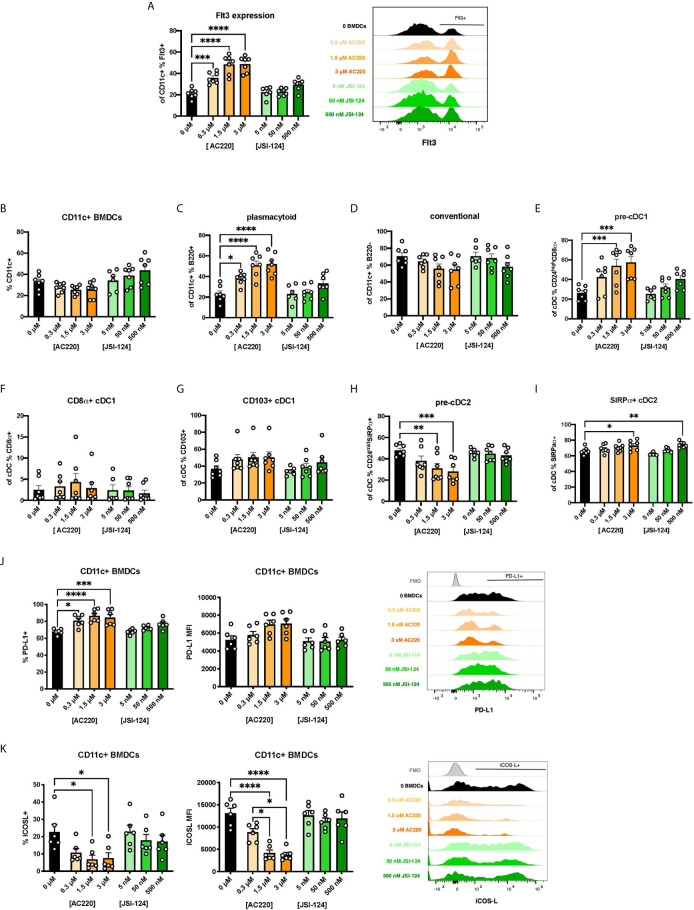
Exposing murine DCs to pharmacological inhibitors of Flt3 and STAT3 phenocopies the effect of BEN exposure. **(A-K)** BALB/c BMDCs were generated following brief exposure to Flt3 inhibitor (ACC20) or STAT3 inhibitor (JSI-124) and characterized by flow cytometry. Data is pooled from 3 independent experiments (n=6-7). **(A)** Mean percent Flt3 expression among CD11c^+^ BMDCs is shown with SEM, and representative histogram (right). **(B-I)** Mean percent with SEM of murine DC lineages including **(B)** total CD11c^+^, **(C)** plasmacytoid DCs, **(D)** conventional DCs, **(E)** pre-cDC1s, **(F)** CD8α^+^ cDC1s, **(G)** CD103^+^ cDC1s, **(H)** pre-cDC2s, and **(I)** SIRPα^+^ cDC2s. **(J)** Mean percent PD-L1 expression (left) and MFI (middle) among CD11c^+^ FL-BMDCs shown with SEM, and representative histograms (right). **(K)** Mean percent ICOS-L expression (left) and MFI (middle) among CD11c^+^ FL-BMDCs shown with SEM, and representative histograms (right). One-way ANOVA and Dunnett’s multiple comparisons test were used to determine significance among groups. *P < 0.05, **P < 0.01, ***P < 0.001, ****P < 0.0001.

There is a paucity of research on the biological mechanisms of action of BEN. However, one report found that BEN inhibits canonical STAT3 signaling (32). STAT3 is one of several known signaling molecules downstream of Flt3 providing essential signals for differentiation, survival, and proliferation (13, 48–51). We hypothesized that, by inhibiting STAT3, BEN interrupts Flt3-STAT3 signaling causing a compensatory upregulation of Flt3 surface expression. To test this, we performed parallel experiments exposing murine BM cells to pharmacological inhibitors of Flt3 (AC220, Quizartinib) and STAT3 (JSI-124, Cucurbitacin I) for 4 hours, washing, and then generating FL-BMDCs. Similar to our observations with BEN exposure, inhibition of Flt3 prior to FL-BMDC generation results in increased expression of Flt3 (**Figure 5A**), with a less prominent trend resulting from STAT3 inhibition. We observed similar DC composition following Flt3 inhibition with a significant increase in pDCs, pre-cDC1s, and SIRPα^+^ cDC2s, and a decrease in pre-cDC2s (**Figures 5B–I**), and a similar trend that was not statistically significant following STAT3 inhibition. We also found similarly increased PD-L1 (**Figure 5J**) and decreased ICOSL expression (**Figure 5K**) following Flt3 inhibition, and to a lesser extent STAT3 inhibition. In support of our hypothesis, exposure to inhibitors of Flt3 and STAT3 phenocopies the effects observed following BEN exposure, with the Flt3 inhibitor showing the most significant response and the STAT3 inhibitor showing slight trends.

### Human moDCs Exposed to BEN Have Increased Flt3 Expression and Decreased pSTAT3

**Figure 6:**
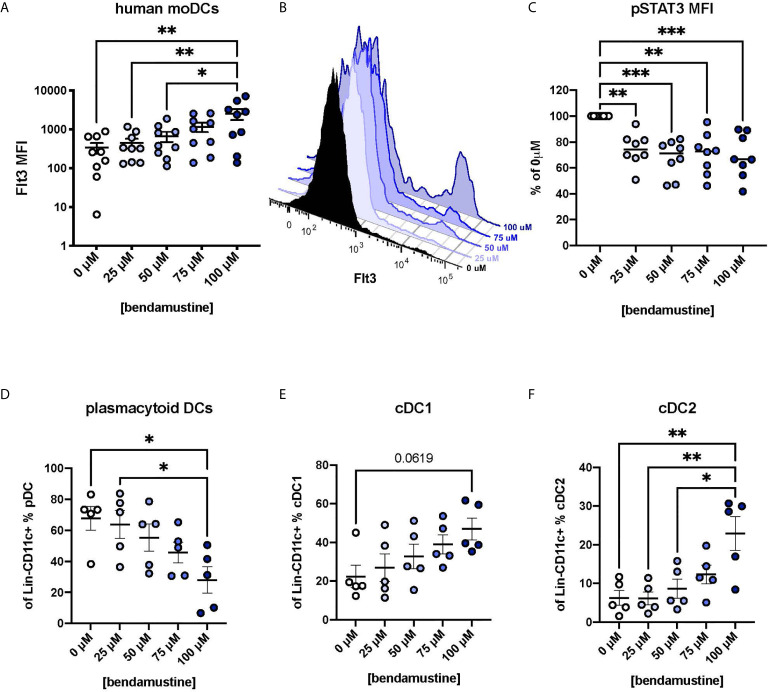
Human DCs exhibit concentration-dependent increase in Flt3 expression following BEN exposure and decreased pSTAT3. Human monocyte-derived DCs (moDCs) were generated following brief exposure to BEN and characterized by flow cytometry. Data shown is pooled from 9 independent experiments (n=5-9). **(A)** Mean Flt3 MFI among human moDCs shown with SEM. **(B)** Representative histogram of Flt3 expression on BEN-exposed moDCs from a single individual. **(C)** Mean pSTAT3 MFI normalized to percent of control (0μM) shown with median. **(D)** Mean percent of plasmacytoid DCs (Lineage^-^CD11c^+^BDCA4^+^) shown with SEM. **(E)** Mean percent of cDC1 (Lineage^-^CD11c^+^BDCA4^-^BDCA3^+^) shown with SEM. **(F)** Mean percent cDC2 (Lineage^-^CD11c^+^BDCA4^-^BDCA1^+^) shown with SEM. One-way ANOVA and Dunnett’s multiple comparisons test were used to determine significance among groups. *P < 0.05, **P < 0.01, ***P < 0.001.

Finally, we wanted to determine whether BEN similarly affects human DCs and if so, if those effects are Flt3-STAT3-mediated. We isolated CD14^+^ monocytes from healthy volunteers to generate moDCs according to established protocols (35–37). Monocytes were exposed to various concentrations of BEN for 4 hours, washed, and moDCs were generated. moDCs exhibited a concentration-dependent increase in Flt3 expression (**Figure 6A**) shown in representative histograms (**Figure 6B**). We also found that these moDCs had significantly decreased phospho-STAT3 (**Figure 6C**). Further studies were conducted to look at DC subsets and found that BEN exposure did not affect moDC purity **(Supplementary Figures 5A, B)** and resulted in a decreased percent of pDCs (**Figure 6D**), a trend toward increased cDC1s (**Figure 6E**), and increased cDC2s (**Figure 6F**). We additionally found small increases in the expression of DNGR1 **(Supplementary Figure 5C)**, another marker for cDC1s, and AXL **(Supplementary Figure 5D)**, a receptor that suppresses inflammatory signaling and limits expression of pro-inflammatory cytokines (52, 53). Consistent with our hypothesis, moDCs exposed to BEN exhibit increased Flt3 expression, decreased pSTAT3, and altered DC composition.

### Murine and Human DCs Exhibit Decreased Akt1 Transcripts as the Concentration of BEN Increases

Molecular work to determine whether Flt3-STAT3 signaling is altered was largely inconclusive, with inconsistent changes in transcript levels of Flt3, STAT3, PU.1, Csfr2a, Csf2rb, and Csf3r **(Supplementary Figure 6A)**. Given the sustained inhibition of pSTAT3 in moDCs, we investigated alternative signaling pathways downstream of Flt3 and found that transcript levels of Akt1 were significantly decreased in murine FL-BMDCs **(Supplementary Figure 6B)** and human moDCs **(Supplementary Figure 6C)** exposed to BEN. Protein levels of phosphorylated-Akt1 were largely undetectable in moDC samples making it difficult to make conclusions about the signaling events downstream of Flt3.

## Discussion

Graft-versus-host disease remains a significant obstacle to the success of HSCT. Administration of Flt3L *prior* to murine BMT significantly improves GvHD through effects on host DCs (22, 23). Our laboratory has demonstrated that replacing cyclophosphamide with bendamustine, both supplemented with TBI, as a pre-transplant conditioning regimen significantly improves GvHD while maintaining GvL in a murine major-mismatch BMT model (25–27). Extensive investigation of various immune populations following these conditioning regimens found that BEN+TBI conditioning yields donor T-cells that are tolerant to host MHC antigens, yet remain reactive to third-party MHC antigens, while preserving T-cell-dependent GvL (26). We have also reported that BEN+TBI results in robust accumulation of host pre-cDC1s, as well as increased Flt3 expression on host DCs (27). In line with enhanced Flt3 signaling (49), we have reported increased number and suppressive function of myeloid-derived suppressor cells (MDSCs) with BEN+TBI conditioning (25). The biological implications of increased Flt3 expression on DCs are not well understood, and to our knowledge the role of pre-cDC1s in alloreactivity and GvHD has not been previously published.

Our results indicate that BEN increases Flt3 expression in a dose-dependent manner *in vivo* on murine cells, and a concentration-dependent manner *in vitro* in both murine and human cells. We report that BEN-exposure favors the development of murine pDCs, pre-cDC1s, and cDC2s, though further studies would be required to determine whether Flt3 over-expression is responsible for deviations in DC lineage commitment. Increased pDCs and pre-cDC1s were also found in our previous studies with *in vivo* administration of BEN. Of note, concentrations used in our present *in vitro* studies encompass physiological levels reached approximately 2 hours after administration of BEN. While there is no pre-cDC1 equivalent identified in humans, we similarly observed increased cDC2s and a trend toward increased cDC1s, though they differ from murine studies in that we observed a decrease in pDCs. This divergence may be due to the inherent nature of the protocol in that monocytes are exposed to BEN, as opposed to bone marrow cells. We must also note that we did not distinguish monocytic-DCs (Lineage-CD11c^+^CD16^+^) in our phenotyping studies. Nevertheless, enhanced Flt3 expression with BEN exposure was consistent between murine and human DCs.

Importantly, administration of Flt3L to the donor does not modify GvHD, and administration of Flt3L to the recipient *post*-transplant accelerates GvHD lethality (21). Further, *in vitro* studies comparing BMDCs generated with Flt3L versus GM-CSF have consistently observed that Flt3L-driven BMDCs are much more steady state-like, producing fewer pro-inflammatory cytokines and inducing less T-cell proliferation (42–44). This body of work suggests that enhancing Flt3 signaling with exogenous Flt3L, specifically among host DCs, results in regulatory DCs that limit alloreactive T-cell responses, are less pro-inflammatory, and prevent GvHD. We posit that this GvHD-suppressing phenotype may extend to our findings with BEN, whereby Flt3 signaling is enhanced *via* increased Flt3 receptor expression on host DCs, rather than with Flt3L administration.

Pre-transplant conditioning regimen components (e.g. total body irradiation) disrupt epithelial barrier integrity and allow translocation of microbial products, such as LPS. GvHD is significantly exacerbated by inflammation caused by recognition of LPS, whereas LPS antagonism has been found to suppress GvHD (54, 55). Using LPS stimulation as a surrogate for total body irradiation experienced *in vivo*, we demonstrate that BEN exposure induces FL-BMDCs that are minimally responsive to LPS. It should be noted that unstimulated FL-BMDCs previously exposed to 100μM of BEN expressed greater percent CD80 and CD86 at baseline, however MFIs were comparable, and upon LPS stimulation exhibited no further increase. FL-BMDCs exposed to 100μM of BEN were found to secrete extremely low levels of pro-inflammatory cytokines and chemokines linked to GvHD development (IL-6, TNFα, CCL2, CCL5, and IL-12p40) (56–59). BEN-exposed BMDCs showed no deficit in IL-10 secretion and no evidence of diminished intracellular levels of these cytokines. This suggests that the phenotype induced by BEN exposure is associated with a suppressed pro-inflammatory response to LPS that may contribute to BEN’s protective effect on GvHD.

The outcome of alloreactivity is ultimately determined by the orchestra of co-signaling molecules present during allogeneic T-cell priming (59, 60). We demonstrated a concentration-dependent increase in PD-L1 expression on FL-BMDCs exposed to BEN. PD-L1-mediated inhibitory signaling *via* PD-1 is essential for the induction and maintenance of peripheral tolerance in transplantation (61, 62). T-cells stimulated with 100μM BEN-exposed FL-BMDCs exhibited a striking increase in PD-1 expression and accelerated proliferation, followed by activation-induced death of half of all allogeneic T-cells in culture. The induction of programmed cell death of alloreactive T-cells has been specifically linked to PD-L1 (63) and is critical to induction and maintenance of peripheral tolerance in transplantation (64–67). It is also worth noting that our previous study found that DCs isolated from BEN-treated mice induced less allogeneic T-cell proliferation compared to CY-treated mice. However, in these previous studies, proliferation was determined by tritiated-thymidine uptake, providing a single snapshot of actively proliferating T-cells. As such, our report of reduced T-cell proliferation from day 3 to day 4 of co-culture may be a reflection of increased alloreactive T-cell death induced by BEN-DCs and is in line with our current findings.

Interestingly, Hill’s group has also reported this phenomenon, demonstrating that host CD8α^+^ cDC1s induce the proliferation and subsequent deletion of allogeneic CD8^+^ T-cells, and that this effect is enhanced by Flt3L administration (23). We observe deletion of CD4^+^ T-cells rather than CD8^+^ T-cells, which is in agreement with Hill’s findings since CD8α^+^ cDC1s constitute a very small proportion of DCs in our assay. This suggests that another Flt3L-driven DC population is capable of inducing specific deletion of alloreactive CD4^+^ T-cells while sparing CD8^+^ T-cells, which could potentially preserve GvL responses. We postulate that pre-cDC1s may be responsible for this effect, which may explain why BEN+TBI results in tolerant donor T-cells while maintaining T-cell-dependent, mostly reliant on CD8^+^ T-cells, GvL (26, 27), though we cannot rule out a contribution of cDC2s.

We found that a Flt3 inhibitor closely replicates many of our findings with BEN, while a STAT3 inhibitor induces similar trends but not significantly so. In agreement with others, we observed decreased levels of phosphorylated Tyrosine 705-STAT3 in human moDCs previously exposed to BEN, though we were surprised that exposure to BEN for just four hours on day 0 resulted in sustained inhibition of STAT3 five days later. Tyrosine 705 is the canonical residue used by Iwamoto’s group to determine that BEN binds to and inhibits STAT3, however these studies did not clarify the kinetics of BEN’s inhibition of STAT3, nor did they explore other possible post-translational modifications (32). STAT3 is a highly pleiotropic molecule. For instance, STAT3 activation by IL-6 induces phosphorylation of Tyr640, and is required for the suppression of LPS-induced DC maturation (68, 69). Therefore, while BEN inhibits canonical STAT3 signaling *via* phosphorylation of Tyr705, we cannot rule out the possibility that STAT3 may still be activated *via* other post-translational modifications resulting in non-canonical STAT3 activation.

Additionally, Flt3L is sufficient and indispensable for the commitment of progenitors to the committed DC progenitor (CDP) stage of DC development, a commitment step that reportedly requires STAT3 (13). However, others have reported that various Flt3L-mediated DC lineage commitment steps alternatively require PI3K, Akt, and mTORC (9, 48). Activation of Akt1/PI3K/mTOR downstream of Flt3 has been shown to play an essential role in regulating lifespan, pro-inflammatory cytokine production, and autophagy in DCs (33, 48, 51, 70–72). In recent years, the regulation of autophagy in DCs has been shown to affect long-term storage and cross-presentation of antigen and critically determine GvHD and GvL effects (33, 73–75). We found a concentration-dependent decrease in transcript levels of Akt1 in both murine FL-BMDCs and human moDCs. This may indicate that Akt1 transcripts were translated into protein by day 6 of culture, however we were unable to measure protein levels of Akt1 to test this. While our current studies do not clearly define the signaling mechanisms associated with BEN exposure, they suggest differential modulation of the signaling events downstream of Flt3. Additionally, the phenotype we observe here closely resembles that of Flt3L-driven BMDCs, supporting the overarching hypothesis that BEN elicits these effects in DCs by positively modulating the Flt3 signaling pathway.

In summary, we demonstrated that bendamustine directly increases Flt3 expression on murine and human DCs and affects DC ontogeny. BEN-exposure and enhanced Flt3 expression are associated with a distinct semi-mature phenotype in murine FL-BMDCs, with greater CD80 and CD86 expression, but increased PD-L1 expression and dampened cytokine response to LPS stimulation. These regulatory FL-BMDCs induced robust proliferation of alloreactive CD4^+^ T-cells followed by programmed cell death. This effect may be attributable to pre-cDC1s and appears to spare CD8^+^ T-cells, providing a potential mechanism by which BEN+TBI conditioning limits GvHD and yields donor T-cells that are tolerant to host antigen while maintaining T-cell-dependent GvL (26).

## Data Availability Statement

The raw data supporting the conclusions of this article will be made available by the authors, without undue reservation.

## Ethics Statement

The studies involving human participants were reviewed and approved by The University of Arizona’s Institutional Review Board. The patients/participants provided their written informed consent to participate in this study. The animal study was reviewed and approved by The University of Arizona, Institutional Animal Care and Use Committee.

## Author Contributions

MM designed and performed experiments, analyzed and reviewed data, and wrote the manuscript. EH and JS helped design and perform experiments, reviewed data, and revised the manuscript. NK, KS, FB, and TZ performed experiments and analyzed data. RS contributed to the experimental design, data interpretation and discussion and revised the manuscript. EK supervised and advised on the implementation and conduction of experiments, reviewed and interpreted data, and co-wrote the manuscript. All authors contributed to the article and approved the submitted version.

## Conflict of Interest

The authors declare that the research was conducted in the absence of any commercial or financial relationships that could be construed as a potential conflict of interest.
