# Supplementary material for: Regulatory Dendritic Cells Induced by Bendamustine Are Associated With Enhanced Flt3 Expression and Alloreactive T-Cell Death
Source: Front Immunol. 2021 Jun 24;12:699128. doi: 10.3389/fimmu.2021.699128 (PMC8264365; doi:10.3389/fimmu.2021.699128)
Supplement: Supplementary file 1 [file DataSheet_1.pdf]

# *Supplementary Material*

## Supplemental Figures

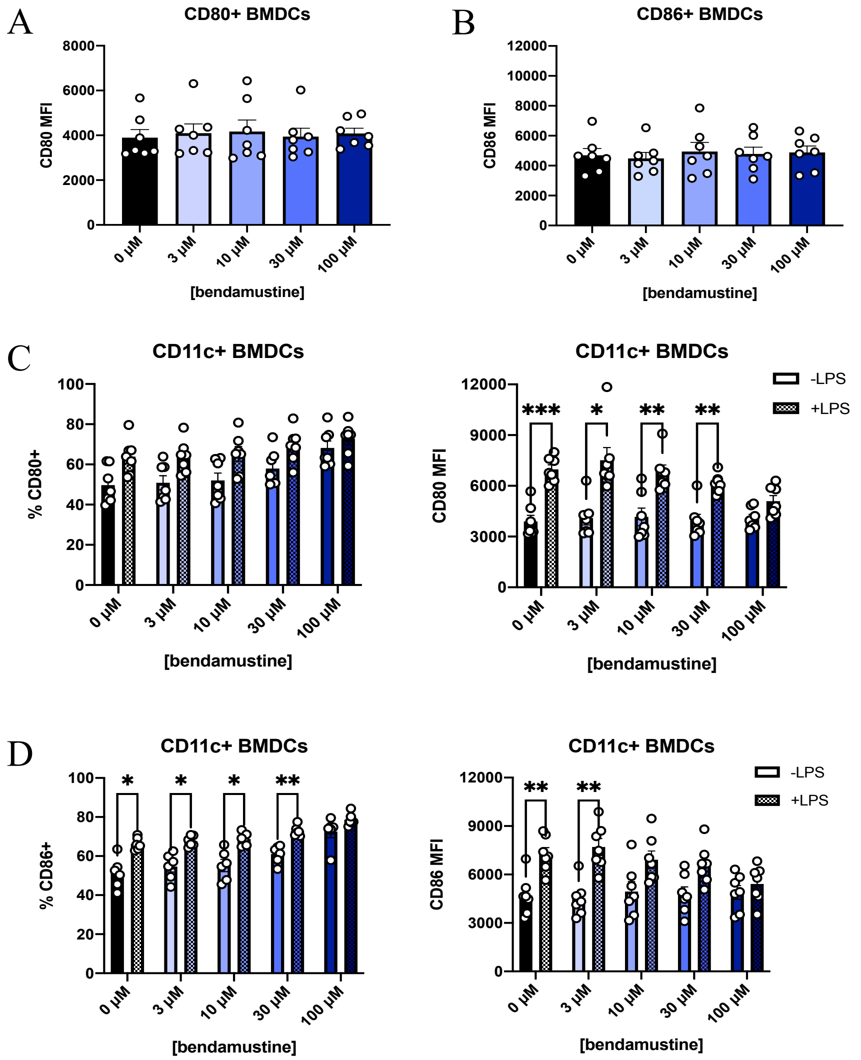


**Supplemental Figure 1.** BALB/c FL-BMDCs were generated following a 4-hour exposure to increasing concentrations of BEN and then characterized by flow cytometry. Data is pooled from 3 independent experiments (n=6-7). (**A**) Mean MFI of CD80 expression on $CD80^+CD11c^+$ FL-BMDCs are shown with SEM. (**B**) Mean MFI of CD86 expression on $CD86^+CD11c^+$ FL-BMDCs are shown with SEM. (**C**) Mean CD80 expression by percent (left) and MFI (right) on $CD11c^+$ FL-BMDCs with or without LPS stimulation are shown with SEM. (**D**) Mean CD86 expression by percent (left) and MFI (right) on $CD11c^+$ FL-BMDCs with or without LPS stimulation are shown with SEM. Two-way ANOVA and Šidák's multiple comparisons test were used to determine significance among groups. *P<0.05, **P<0.01, ***P<0.001

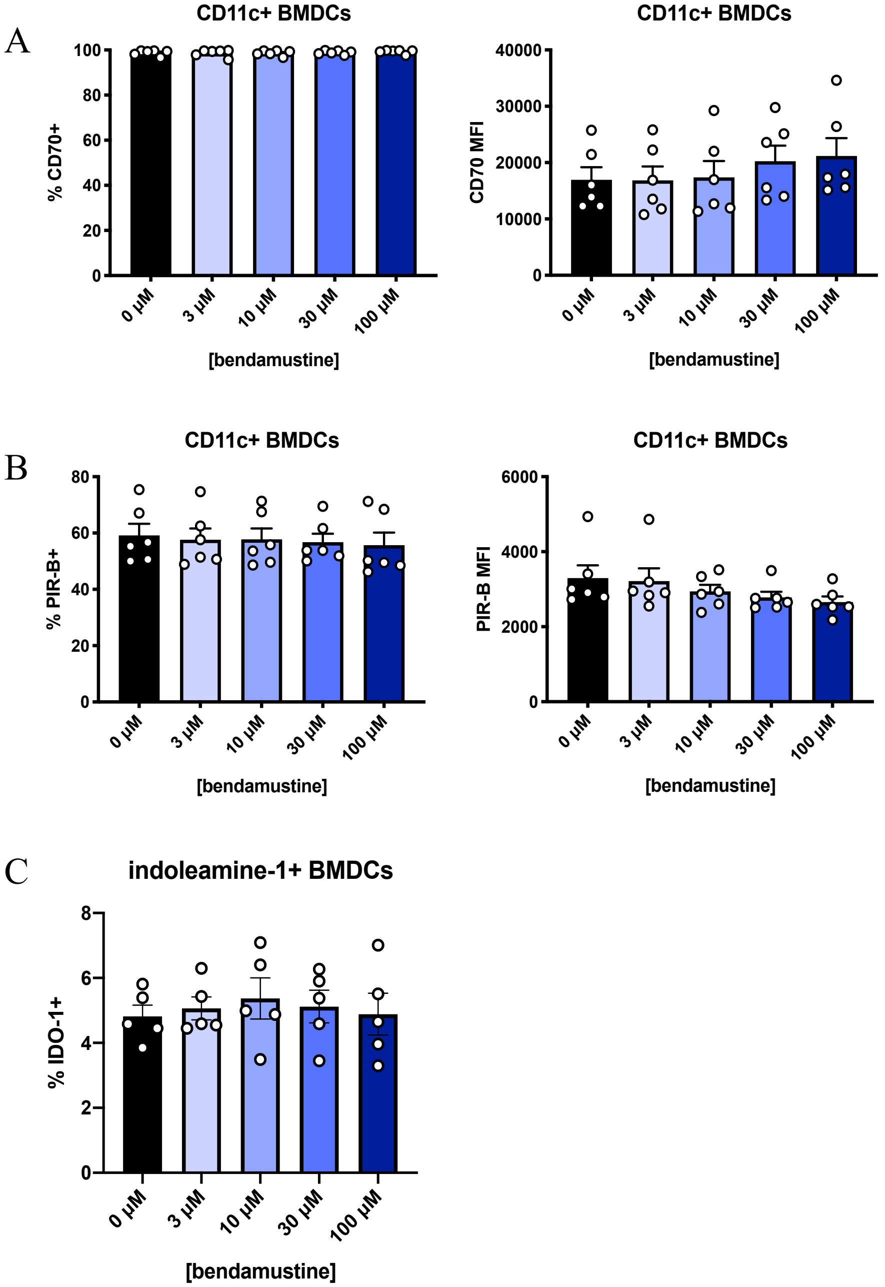


**Supplemental Figure 2.** BALB/c FL-BMDCs were generated following a 4-hour exposure to increasing concentrations of BEN and then characterized by flow cytometry. Data is pooled from 3 independent experiments (n=6-7). (**A**) Mean percent (left) and MFI (right) of CD70 expression on CD11c$^+$ FL-BMDCs are shown with SEM. (**B**) Mean percent (left) and MFI (right) of PIR-B expression on CD11c$^+$ FL-BMDCs are shown with SEM. (**C**) Mean percent expression of IDO-1 by CD11c$^+$ FL-BMDCs is shown with SEM. One-way ANOVA and Dunnett's multiple comparisons test were used to determine significance among groups.

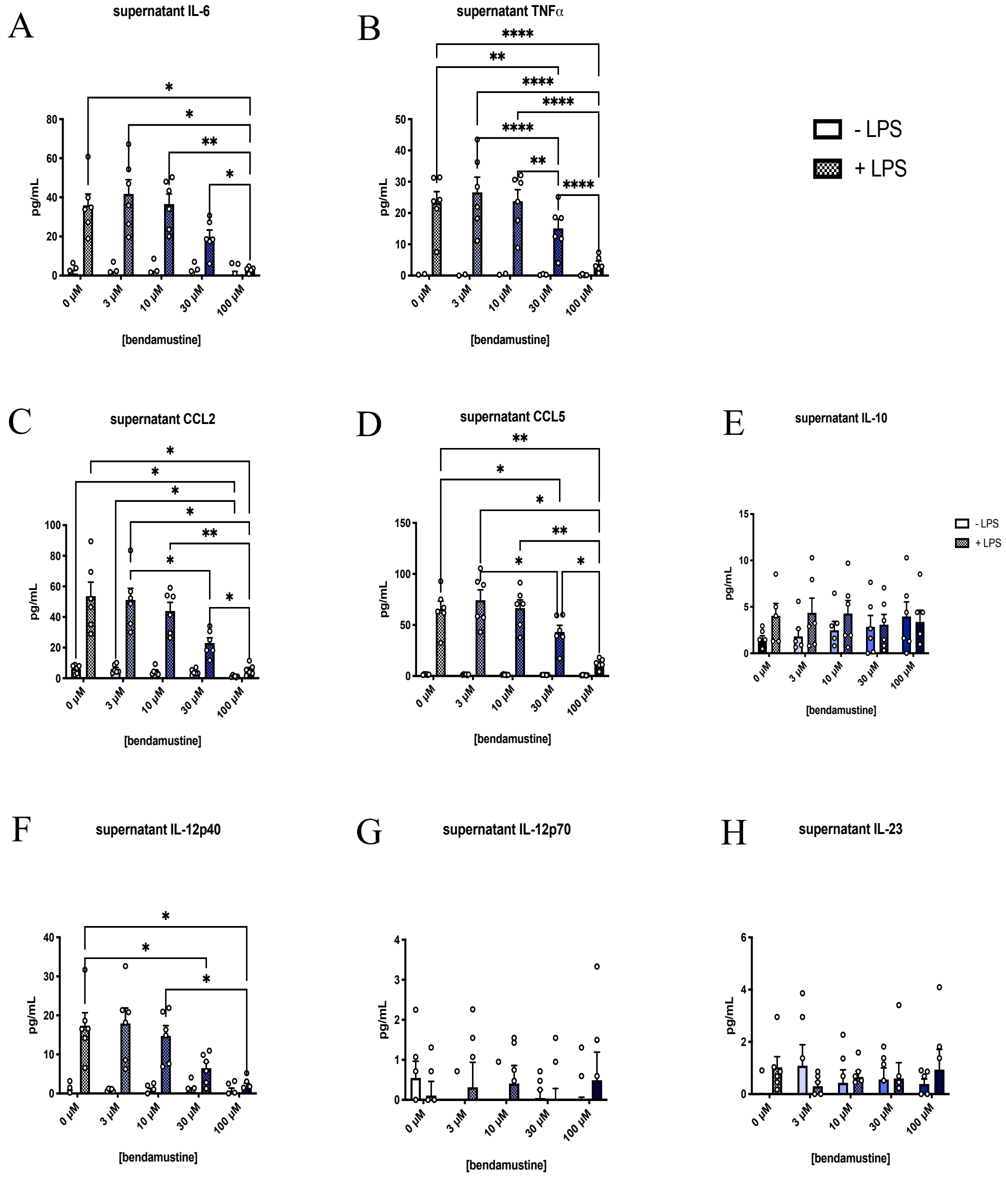


**Supplemental Figure 3.** Statistical comparisons between the different concentrations of BEN shown for the same data in Figure 3. (**A-H**) BALB/c FL-BMDCs were generated following brief exposure to BEN. With or without 18 hours of LPS stimulation, supernatants were collected for ELISA analysis. Data is pooled from 2 independent experiments (n=6). Some values fall below zero, outside of the detectable limits of the assay and outside the axis limits. Mean concentration of (**A**) IL-6, (**B**) TNFα, (**C**) CCL2 (MCP-1), (**D**) CCL5 (RANTES), (**E**) IL-10, (**F**) IL-12p40, (**G**) IL-12p70, and (**H**) IL-23 in supernatants is shown with SEM. Two-way ANOVA and Šidák's multiple comparisons test were used to determine significance among groups. $^*P<0.05$, $^{**}P<0.01$, $^{****}P<0.0001$

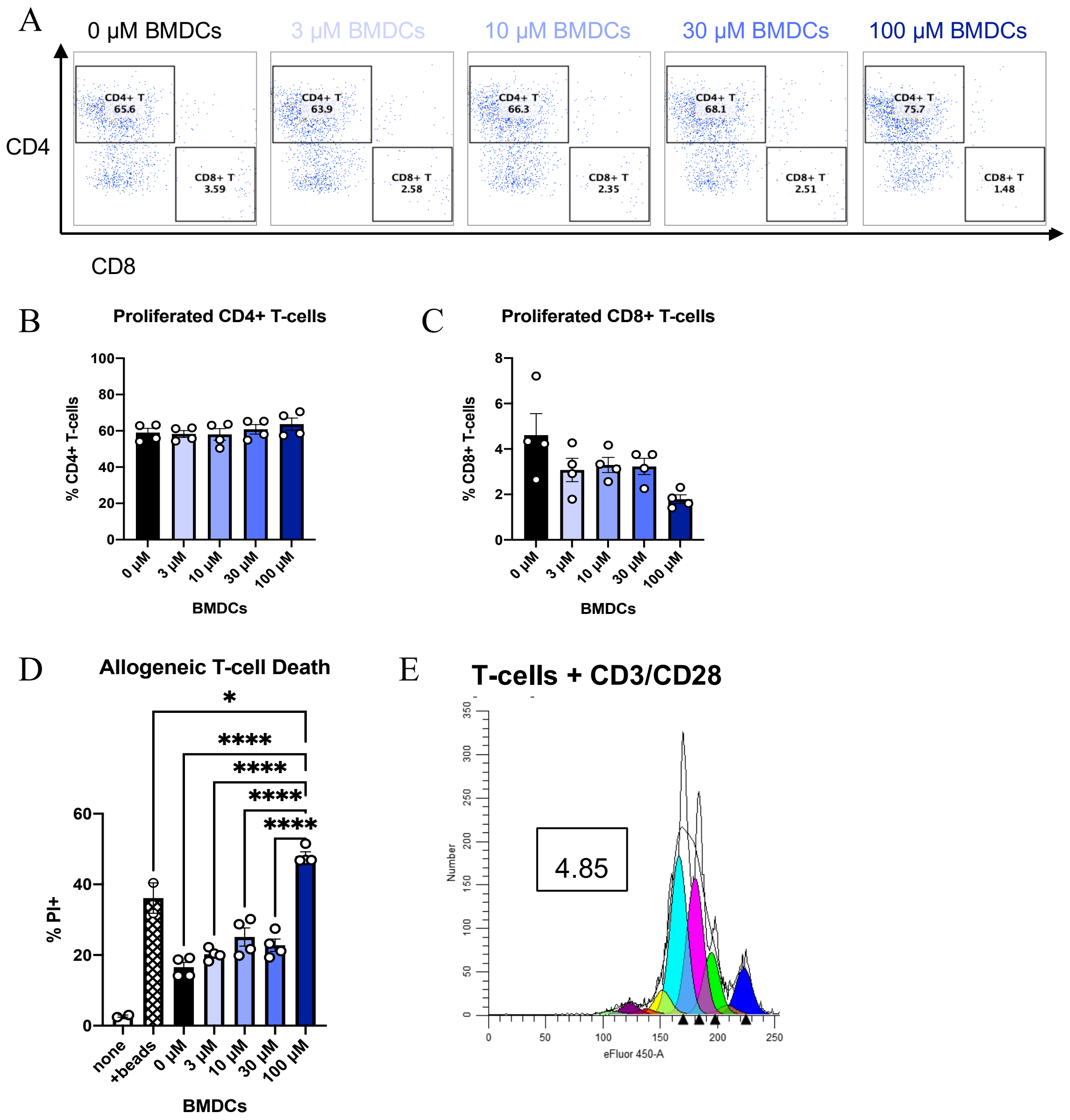


**Supplemental Figure 4.** BALB/c FL-BMDCs were generated following a 4-hour exposure to increasing concentrations of BEN and then used in a mixed leukocyte reaction (MLR) with C57BL/6 CellTrace-stained, allogeneic T-cells. Data is representative of 2 independent experiments (n=4). (**A**) Representative flow cytometry plots indicating the percentage of $CD4^+$ and $CD8^+$ T-cells in the proliferative fraction (Gated on $H2K^{b+}CellTrace^{low}$) on day 3 of the assay. (**B**) Mean percent $CD4^+$ T-cells shown with SEM. (**C**) Mean percent $CD8^+$ T-cells shown with SEM. (**D**) Mean percent death ($PI^+$) of T-cells on day 4 is shown with SEM. (**E**) Representative histogram produced by ModFit software depicted T-cell proliferation induced by CD3/CD28 beads, quantified by proliferation index (boxed value). One-way ANOVA and Dunnett's multiple comparisons test were used to determine significance among groups. *$P<0.05$, ****$P<0.0001$

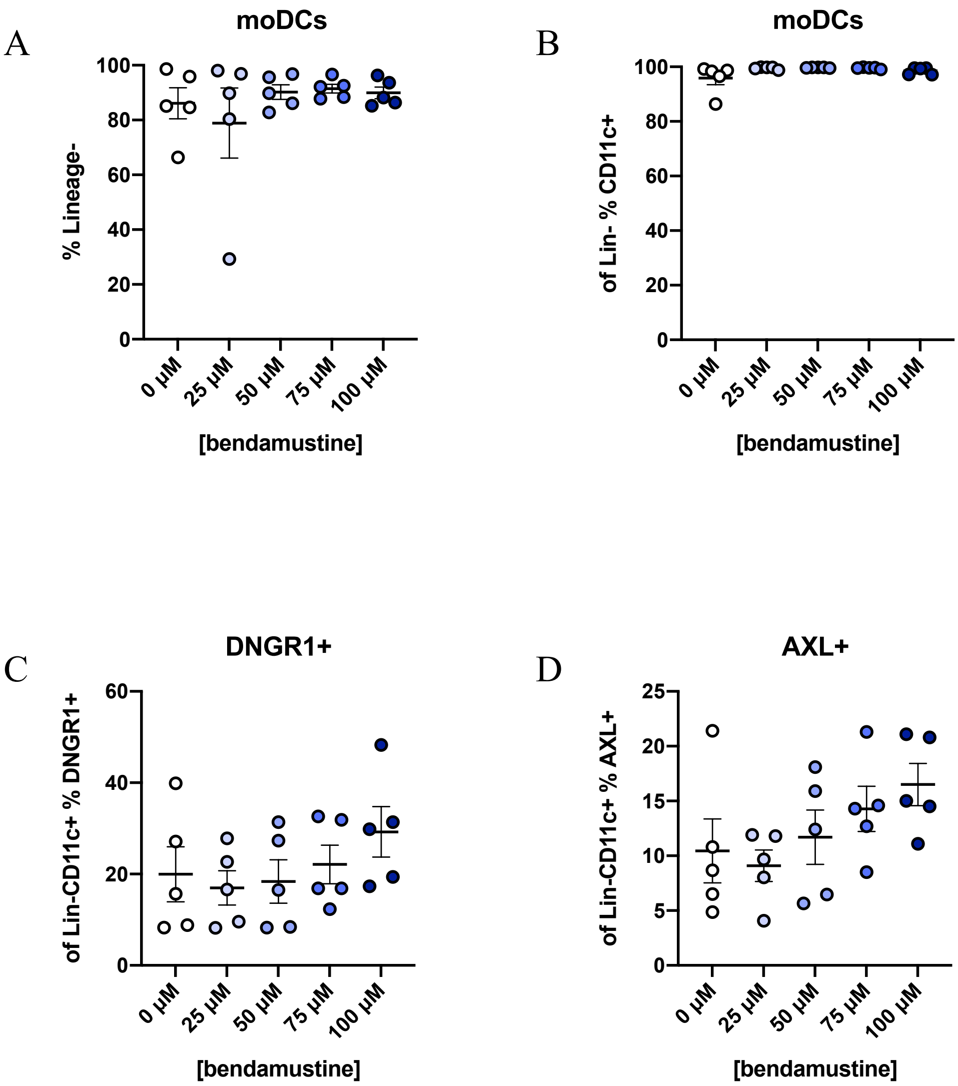


**Supplemental Figure 5.** Human moDCs were generated following a 4-hour exposure to increasing concentrations of BEN and then characterized by flow cytometry. Data is pooled from 3 independent experiments (n=5). (**A**) Mean percent Lineage$^-$ moDCs shown with SEM. (**B**) Mean percent CD11c$^+$ moDCs among Lineage- shown with SEM. (C) Mean percent DNGR1$^+$ among Lin-CD11c$^+$ moDCs shown with SEM. (D) Mean percent AXL$^+$ moDCs among Lin-CD11c$^+$ moDCs shown with SEM. One-way ANOVA and Dunnett's multiple comparisons test were used to determine significance among groups.

A

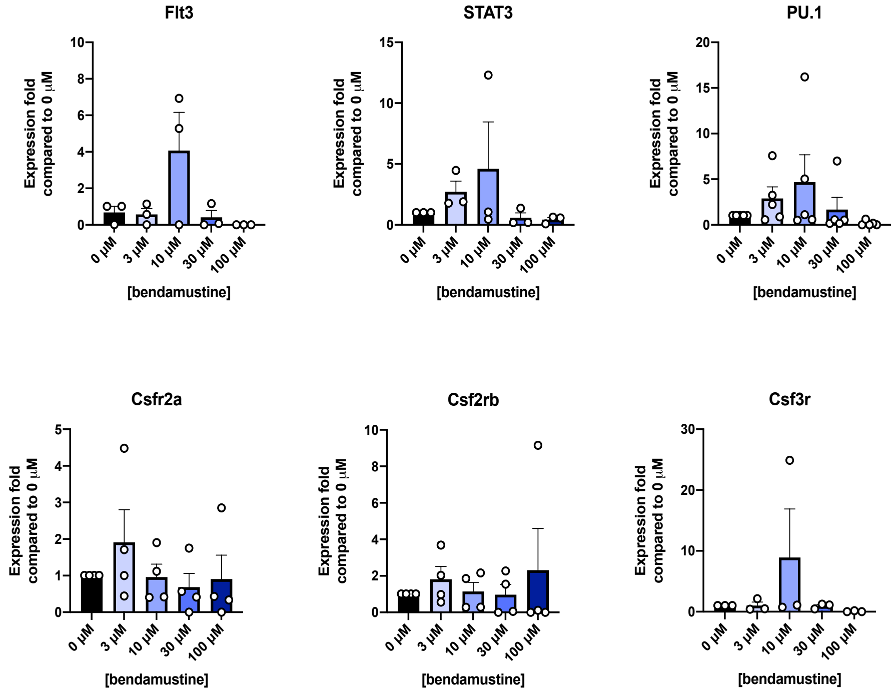


B

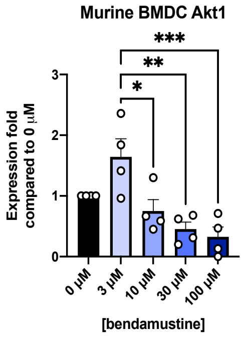


C

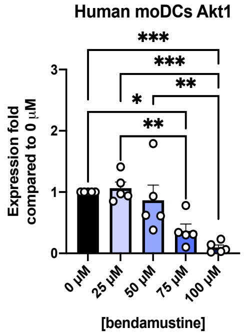


**Supplemental Figure 6.** (**A-B**) BALB/c FL-BMDCs were generated following a 4-hour exposure to increasing concentrations of BEN and then used for quantitative rtPCR analysis. Data is pooled from of 2 independent experiments (n=6). (**A**) Mean fold change of gene expression of FL-BMDCs with SEM is shown, normalized to GAPDH. (**B**) Mean fold change of murine Akt1 gene expression of FL-BMDCs with SEM is shown, normalized to GAPDH. (**C**) Mean fold change of human Akt1 gene expression of human moDCs with SEM is shown, normalized to GAPDH. One-way ANOVA and Dunnett's multiple comparisons test were used to determine significance among groups. *P<0.05, **P<0.01, ***P<0.001
